# The Short-Term Impact of Ontario's Generic Pricing Reforms

**DOI:** 10.1371/journal.pone.0023030

**Published:** 2011-07-28

**Authors:** Michael R. Law, Alison Ystma, Steven G. Morgan

**Affiliations:** The Centre for Health Services and Policy Research, School of Population and Public Health, The University of British Columbia, Vancouver, British Columbia, Canada; Canadian Agency for Drugs and Technologies in Health, Canada

## Abstract

**Background:**

Canadians pay amongst the highest generic drug prices in the world. In July 2010, the province of Ontario enacted a policy that halved reimbursement for generic drugs from the public drug plan, and substantially lowered prices for private purchases. We quantified the impact of this policy on overall generic drug expenditures in the province, and projected the impact in other provinces had they mimicked this pricing change.

**Methods:**

We used quarterly prescription generic drug dispensing data from the IMS-Brogan CompuScript Audit. We used the price per unit in both the pre- and post-policy period and two economics price indexes to estimate the expenditure reduction in Ontario. Further, we used the post-policy Ontario prices to estimate the potential reduction in other provinces.

**Results:**

We estimate that total expenditure on generic drugs in Ontario during the second half of 2010 was between $181 and $194 million below what would be expected if prices had remained at pre-policy level. Over half of the reduction in spending was due to savings on just 10 generic ingredients. If other provinces had matched Ontario's prices, their expenditures over during the latter half of 2010 would have been $445 million lower.

**Discussion:**

We found that if Ontario's pricing scheme were adopted nationally, overall spending on generic drugs in Canada would drop at least $1.28 billion annually—a 5% decrease in total prescription drug expenditure. Other provinces should seriously consider both changes to their generic drug prices and the use of more competitive bulk purchasing policies.

## Introduction

Canadians pay amongst the highest generic drug prices in the world [1], [2]. For example, a recent comparison found that in 2007 Canada had the highest overall prices for nearly 300 commonly used generic drugs in comparison to 11 other countries [3]. Importantly, the scale of the price differences was large—Canadian prices were more than double those in the United States and over five-fold higher than New Zealand. High Canadian prices are direct consequence of provincial governments' predominant pricing strategy for generic drugs, which sets reimbursement for community generic drug prescriptions using a fixed percentage that has typically been 60% to 70% of the equivalent brand name price [1].

In an attempt to reduce provincial drug plan expenditures, in 2006 Ontario changed the price paid by the public Ontario Drug Benefit plan (ODB) to 50% of the brand reference price. In 2010, the Ontario government legislated further generic price reductions [4]. Effective July 2010, the ODB price for generics would be set at 25% of the brand and the private price for generics would be limited to 50% of the brand equivalent immediately. The legislation further required that the private price of generics would drop to 25% by April 2012. These changes provoked a vocal outcry from pharmacies that claimed the lost revenue would have to reduce stores and services. While other provinces continue to assess their options and wait to see the outcome in Ontario, many continue to price generic drugs at the old 60% to 70% level [5].

To inform these decisions and future policies, we investigated the impact of the Ontario policy from two perspectives. First, we assessed the change in expenditures that Ontario experienced over the first six months of their policy. Second, we estimated the potential expenditure reductions other provinces would have experienced if they had mimicked Ontario's pricing strategy.

## Methods

### Data Sources

We obtained quarterly data from 2010 on the number of units (e.g. tablets), prescriptions and total cost for all prescription drugs dispensed from the IMS-Brogan CompuScript audit. These data are drawn from a national panel of over 5,000 pharmacies (approximately 60% of all the community pharmacies in Canada), and are projected to estimate provincial dispensing totals [6]. These data include all prescription drugs paid for by public and private drug plans and through out-of-pocket purchases.

We used an identifier provided by IMS-Brogan to select all generic drugs. As generic prices are set per unit by the ODB, we calculated the pre-policy price-per-unit (PPU) for every generic product in Ontario using data from Quarters 1 and 2 (January–June), and for the post-policy period using data from Quarters 3 and 4 (August–December). We also calculated a second PPU for both periods that excluded an estimate of dispensing fees. For this calculation, we used the allowable dispensing fee under the Ontario Drug Benefit multiplied by the total number of volume in the IMS-Brogan data for each generic product. As we could not calculate a pre-policy PPU for generic drugs that were not sold in Quarter 1 or 2, we excluded any generic product not dispensed in either of these quarters.

### Analysis of Policy Impact in Ontario

We estimated the impact of the policy on generic drug spending in Ontario using pre-post measures based on Laspeyres and Paasche economic indexes [7]. The first method compares actual post-period expenditure with what expenditures would have been in the second half of the year if the average price in Quarters 1 and 2 had continued to be used. The second estimate – required because it can be argued that changes in prices might induce changes in consumption – compares actual pre-policy expenditure with what expenditures would have been if the average price in Quarters 3 and 4 had been applied in the first half of the year. Respectively, these methods provide an upper and lower bound on estimated changes in expenditure due to observed changes in prices of generic drugs. We also determined what individual generic drugs constituted the largest portion of the change in expenditure.

### Analysis of Potential Savings in other Provinces

To estimate the potential savings of reducing generic drug prices in other provinces, we multiplied the average Ontario post-policy PPU and the number of units dispensed in each province. We then calculated the potential expenditure reduction by subtracting this total from the observed levels in each province. Finally, to make the figures comparable across jurisdictions, we calculated per capita amounts using population estimates from Statistics Canada [8].

## Results

### Policy impact in Ontario

**Figure 1:**
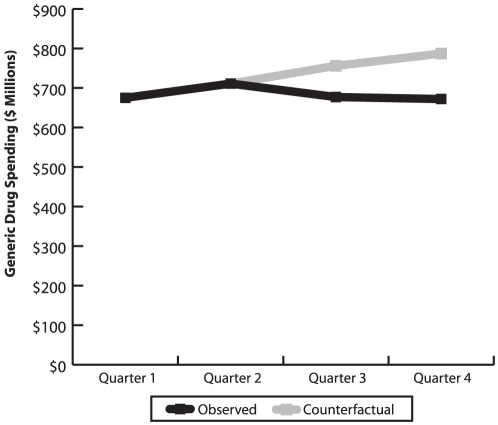
Observed generic spending in Ontario in 2010, and counterfactual estimates derived assuming that the pre-policy per-unit prices continued to be used. Source: IMS Brogan, Canadian CompuScript.

Figure 1 shows actual expenditure on generic drugs in Ontario over the four quarters of 2010 and illustrates the estimated post-policy expenditure in the scenario where prices did not change. Comparing actual and estimated post-policy expenditures indicated that generic drug expenditures in Ontario dropped $194 million due to post-policy changes in generic prices. Constructing the analogous comparison on pre-policy period results in a similar estimate of the change in expenditure due to changes in generic prices: $181 million. In sum, these suggest that annualized generic drug expenditures in Ontario dropped between $362 and $388 million. As expected, when dispensing fees are not included the estimates increased to $240 million and $225 million, respectively.

The top 10 generic ingredients in terms of total change in spending due to changes in price are shown in Table 1. As seen in the table, the cost savings in Ontario were heavily concentrated: $105 million of the estimated $194 million reduction in spending is because of changes in the prices of these 10 generic products. Expenditure reductions for these medicines in 2010 ranged from 11% to 32%, including markups and dispensing fees.

**Table 1:** Top 10 generic products by total change in spending due to changes in price in 2010 in Ontario.

Generic Product	Total Prescriptions (000's)	% Spending Reduction	Estimated change in spending due to changes in price (000's)
Amlodipine	2,714	24.0%	−$14,772
Ramipril	3,264	23.7%	−$14,336
Pantoprazole	1,655	28.3%	−$14,167
Rabeprazole	2,292	28.5%	−$12,166
Venlafaxine	1,674	22.2%	−$10,748
Lansoprazole	1,147	31.3%	−$10,463
Olanzapine	799	32.1%	−$9,857
Omeprazole	989	23.5%	−$8,324
Citalopram	1,967	20.2%	−$7,179
Metformin	3,269	11.4%	−$3,678
**Total**			**−$105,691**

All costs are inclusive of markup and dispensing fees. Source: IMS Brogan, Canadian CompuScript.

### Estimated expenditure changes in other provinces

As shown in Table 2, we found generic drug expenditures in the other provinces would have been approximately $890 million per year lower if they had reimbursed generic drugs at the Ontario price. Further, we also found wide variation in the estimated reductions that other provinces would have realized. On a per-capita basis, our estimated expenditure reductions ranged from an annual savings of $34.69 per capita in BC, to a high of $68.73 in Newfoundland.

**Table 2:** Estimated potential savings in other provinces from matching Ontario generic drug prices.

Province	Generic Exp Q3–Q4 (000's)	Total at Ontario Price Q3–Q4 (000's)	Estimated Potential 6-month Savings (000's)	Estimated Potential Annual Savings (000's)	Potential Annual Savings Per Capita
BC	$476,107	$397,664	$78,443	$156,886	$34.63
Alberta	$437,770	$351,147	$86,623	$173,246	$46.56
Saskatchewan	$120,898	$98,042	$22,855	$45,710	$43.72
Manitoba	$150,763	$122,724	$28,039	$56,078	$45.39
Quebec	$893,357	$727,999	$165,358	$330,716	$41.82
Nova Scotia	$141,365	$119,883	$21,482	$42,964	$45.59
New Brunswick	$121,638	$100,103	$21,534	$43,069	$57.29
PEI	$20,366	$16,758	$3,609	$7,217	$50.73
Newfoundland	$85,966	$68,448	$17,517	$35,035	$68.73
**Total**	**$2,448,230**	**$2,002,770**	**$445,460**	**$890,921**	

All costs are inclusive of markup and dispensing fees. Source: IMS Brogan, Canadian CompuScript.

## Discussion

We found that price changes following the Ontario policy reduced expenditure on generic drugs by between $181 and $194 million over 6 months. Further, we also found that if other provinces mimicked the newer Ontario prices, their expenditures would have been $445 million lower over the same 6 months. In sum, if the Ontario prices applied nationally, the total annual expenditure reduction would be at least $1.28 billion. This represents a 4.9% reduction in total Canadian prescription drug expenditures [9]. Importantly, these savings will only grow as Ontario phases in lower prices for private purchases, and also as more drugs become available as generics.

Our analysis should be considered in light of some limitations. First, the Ontario policy permitted existing stock to be reimbursed at the pre-policy price. However, this would only have acted to make our estimates conservative, as higher prices could continue into the post-policy period. Second, our analysis of other provinces assumes that they would they adopt the Ontario ingredient costs, maximum markup and dispensing fee policies. Unfortunately, comparable data on ingredient costs for all purchasers is not available. However, an analysis by the PMPRB suggests the percentage that provinces spend on professional fees is differs only a small amount: from 16% to 19% of total drug costs [10]. Third, we had to use the Ontario Drug Benefit dispensing fee data to determine the expenditure reductions at the ingredient cost level. Unfortunately, we do not have data on the dispensing fees paid by private plans during these quarters, or information on whether the increased as a result of the policy. Finally, we did not include any “new” generics (those entering in the post-policy period), as we did not have a pre-policy price for comparison. This would also make our estimates conservative, as numerous high-volume drugs continue to become available as generics.

Reductions to generic prices in other provinces would likely face much of the same opposition and concerns that were present in Ontario. In particular, pharmacies would oppose such cuts, claiming they would have to reduce the number of stores. However, prior research has shown that Canada currently has 40% more pharmacies per capita than the US, and prior research has demonstrated that closures in Ontario would only modestly impact geographic access [11]. Moreover, to ensure adequate access to pharmacies in rural areas, policies can be introduced that provide support for these pharmacies, as they were in Ontario.

In summary, Ontario's generic price reductions reduced generic drug expenditure in the province, and could significantly reduce expenditures in every other province. Moreover, these savings will only increase as the Ontario prices continue to drop for private purchases. While these savings are impressive, there is good evidence they could be even larger. It is likely Ontario's prices for many generics remain higher than they might be under a more competitive tendering system [12], [13]. Moving to such a system would remove the arbitrary use of a reference brand price that bears little relation to the actual cost of manufacturing. The money that could be saved on generic drugs large, and on the same scale as the total annual expenditure on Cancer drugs in Canada, or some estimates of the cost of a national catastrophic drug plan [14], [15]. Other provinces should follow Ontario's lead, and governments and private payers should continue to foster competition to lower generic drug prices.
